# (*Z*)-*N*-Ethyl-2-(5-fluoro-2-oxoindolin-3-yl­idene)hydrazinecarbothio­amide

**DOI:** 10.1107/S1600536812036471

**Published:** 2012-09-08

**Authors:** Amna Qasem Ali, Naser Eltaher Eltayeb, Siang Guan Teoh, Abdussalam Salhin, Hoong-Kun Fun

**Affiliations:** a School of Chemical Sciences, Universiti Sains Malaysia, Minden, Penang, Malaysia; b Faculty of Science, Sabha University, Libya; c Department of Chemistry, International University of Africa, Khartoum, Sudan; d X-ray Crystallography Unit, School of Physics,Universiti Sains Malaysia, 11800 USM, Penang, Malaysia

## Abstract

In the title compound, C_11_H_11_FN_4_OS, an intra­molecular N—H⋯O hydrogen bond generates an *S*(6) ring. In the crystal, mol­ecules form chains through N—H⋯O hydrogen bonds, which are extended by N—H⋯S hydrogen bonds into an infinite three-dimensional network.

## Related literature

For related structures, see: Ali *et al.* (2012*a* ▶,*b* ▶); Qasem Ali *et al.* (2011 ▶; 2012**a* ▶,b* ▶,*c* ▶,*d* ▶). For graph-set analysis, see Bernstein *et al.* (1995 ▶). For the biological activity of isatin and its derivatives, see: Suryavanshi & Pai (2006 ▶); Pandeya *et al.* (1999 ▶); Bhandari *et al.* (2008 ▶).
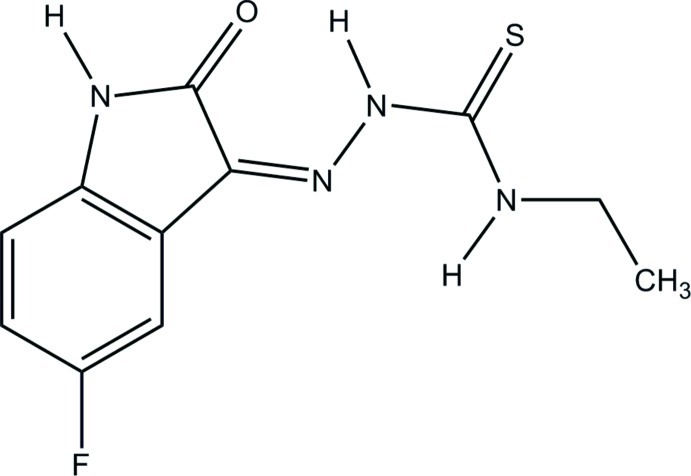


## Experimental

### 

#### Crystal data

C_11_H_11_FN_4_OS
*M* *_r_* = 266.30
Orthorhombic, 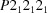

*a* = 4.5151 (1) Å
*b* = 11.6102 (3) Å
*c* = 22.3255 (7) Å
*V* = 1170.33 (5) Å^3^
*Z* = 4
Mo *K*α radiation
μ = 0.28 mm^−1^
*T* = 100 K
0.43 × 0.09 × 0.05 mm

#### Data collection

Bruker APEXII CCD diffractometer
Absorption correction: multi-scan (*SADABS*; Bruker, 2005 ▶) *T*_min_ = 0.887, *T*_max_ = 0.987
8163 measured reflections
3384 independent reflections
2558 reflections with *I* > 2σ(*I*)
*R*_int_ = 0.047

#### Refinement

*R*[*F* ^2^ > 2σ(*F* ^2^)] = 0.064
*wR*(*F* ^2^) = 0.098
*S* = 1.09
3384 reflections
176 parameters
H atoms treated by a mixture of independent and constrained refinement
Δρ_max_ = 0.48 e Å^−3^
Δρ_min_ = −0.40 e Å^−3^
Absolute structure: Flack (1983 ▶), 1367 Friedel pairs
Flack parameter: −0.12 (12)

### 

Data collection: *APEX2* (Bruker, 2005 ▶); cell refinement: *SAINT* (Bruker, 2005 ▶); data reduction: *SAINT*; program(s) used to solve structure: *SHELXS97* (Sheldrick, 2008 ▶); program(s) used to refine structure: *SHELXL97* (Sheldrick, 2008 ▶); molecular graphics: *SHELXTL* (Sheldrick, 2008 ▶); software used to prepare material for publication: *SHELXTL* and *PLATON* (Spek, 2009 ▶).

## Supplementary Material

Crystal structure: contains datablock(s) I, global. DOI: 10.1107/S1600536812036471/fy2062sup1.cif

Structure factors: contains datablock(s) I. DOI: 10.1107/S1600536812036471/fy2062Isup2.hkl

Supplementary material file. DOI: 10.1107/S1600536812036471/fy2062Isup3.cml

Additional supplementary materials:  crystallographic information; 3D view; checkCIF report

## Figures and Tables

**Table 1:** Hydrogen-bond geometry (Å, °)

*D*—H⋯*A*	*D*—H	H⋯*A*	*D*⋯*A*	*D*—H⋯*A*
N1—H1*N*1⋯O1^i^	0.84 (2)	2.01 (2)	2.836 (3)	168 (3)
N3—H1*N*3⋯O1	0.88 (2)	2.06 (2)	2.747 (3)	134 (2)
N4—H1*N*4⋯S1^ii^	0.80 (3)	2.86 (2)	3.541 (2)	144 (2)

Symmetry codes: (i) 
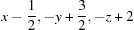
; (ii) 
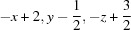
.

**Fig. 1.**
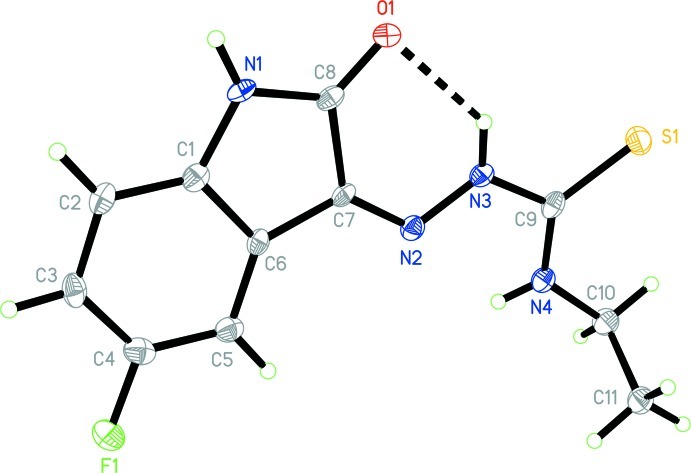
Molecular structure of the title compound, with 50% probability displacement ellipsoids and the atom numbering scheme.

**Fig. 2.**
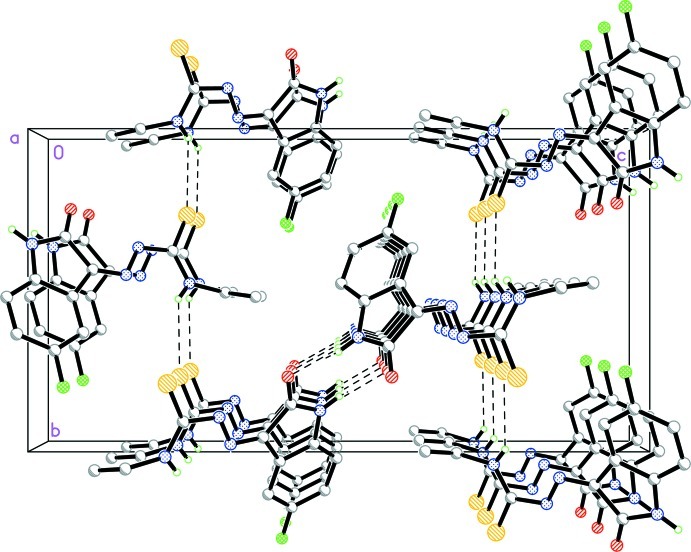
Crystal packing of the title compound viewed down the a axis. Hydrogen bonds are shown as dashed lines.

## supplementary crystallographic information

### Comment

Isatin (2,3-dioxindole) is an endogenous compound identified in humans, and its effect has been studied in a variety of systems. The biological properties of isatin and its derivatives include a range of actions in the brain, they offer protection against bacterial (Suryavanshi & Pai, 2006) and fungal infections and they confer anticonvulsant, anti-HIV (Pandeya *et al.*, 1999), anti-depressant and anti-inflammatory activities (Bhandari *et al.*, 2008). In the present paper we describe the single-crystal X-ray diffraction study of title compound, Fig. 1.

In the title compound C_11_H_11_FN_4_OS, (Fig. 1), the intramolecular N3—H1N3···O1 hydrogen-bonding interaction generates a ring motif [graph set *S*(6)]. In the crystal the molecules form chains through intermolecular N1—H1N1···O1 hydrogen bonds, which are extended by the N4—H1N4···S1 hydrogen bonding interaction into an infinite three-dimensional network (Fig. 2, Table 1).

### Experimental

The Schiff base has been synthesized by refluxing the reaction mixture of a hot ethanolic solution (30 ml) of 4-ethyl-3-thiosemicarbazide (0.01 mol) and a hot ethanolic solution (30 ml) of 5-flouroisatin (0.01 mol) for 2 hrs. The precipitate formed on cooling of the reaction mixture was filtered, washed with cold EtOH and recrystallized from hot EtOH. Yield: 80%; m.p.: 521.3–522.1 K. The yellow crystals were grown in ethanol-DMF (4:1) by slow evaporation at room temperature.

### Refinement

N-bound H atoms were located in a difference Fourier map and were refined freely. The remaining H atoms were positioned geometrically and refined using a riding model, with C—H = 0.95 Å for aromatic ring, C—H = 0.98 Å for methyl group and C—H = 0.99 Å for methylene group H atoms, and with *U*_iso_(H) = 1.2 U_eq_(C), 1.2U_eq_(C) and 1.5U_eq_(C) for aromatic ring, methylene group and methyl group H atoms, respectively. The highest residual electron density peak is located at 0.11 Å from S1 and the deepest hole is located at 0.47 Å from S1.

### Crystal data

**Table:**

C_11_H_11_FN_4_OS	*D*_x_ = 1.511 Mg m^−^^3^
*M**_r_* = 266.30	Melting point = 521.3–522.1 K
Orthorhombic, *P*2_1_2_1_2_1_	Mo *K*α radiation, λ = 0.71073 Å
Hall symbol: P 2ac 2ab	Cell parameters from 1888 reflections
*a* = 4.5151 (1) Å	θ = 3.3–26.6°
*b* = 11.6102 (3) Å	µ = 0.28 mm^−^^1^
*c* = 22.3255 (7) Å	*T* = 100 K
*V* = 1170.33 (5) Å^3^	Needle, yellow
*Z* = 4	0.43 × 0.09 × 0.05 mm
*F*(000) = 552	

### Data collection

**Table:**

Bruker APEXII CCD diffractometer	3384 independent reflections
Radiation source: fine-focus sealed tube	2558 reflections with *I* > 2σ(*I*)
Graphite monochromator	*R*_int_ = 0.047
φ and ω scans	θ_max_ = 30.0°, θ_min_ = 1.8°
Absorption correction: multi-scan (*SADABS*; Bruker, 2005)	*h* = −6→3
*T*_min_ = 0.887, *T*_max_ = 0.987	*k* = −16→16
8163 measured reflections	*l* = −31→19

### Refinement

**Table:**

Refinement on *F*^2^	Secondary atom site location: difference Fourier map
Least-squares matrix: full	Hydrogen site location: inferred from neighbouring sites
*R*[*F*^2^ > 2σ(*F*^2^)] = 0.064	H atoms treated by a mixture of independent and constrained refinement
*wR*(*F*^2^) = 0.098	*w* = 1/[σ^2^(*F*_o_^2^) + (0.0233*P*)^2^ + 0.3031*P*] where *P* = (*F*_o_^2^ + 2*F*_c_^2^)/3
*S* = 1.09	(Δ/σ)_max_ = 0.001
3384 reflections	Δρ_max_ = 0.48 e Å^−^^3^
176 parameters	Δρ_min_ = −0.40 e Å^−^^3^
0 restraints	Absolute structure: Flack (1983), 1367 Friedel pairs
Primary atom site location: structure-invariant direct methods	Flack parameter: −0.12 (12)

### Special details

**Table:**

Geometry. All e.s.d.'s (except the e.s.d. in the dihedral angle between two l.s. planes) are estimated using the full covariance matrix. The cell e.s.d.'s are taken into account individually in the estimation of e.s.d.'s in distances, angles and torsion angles; correlations between e.s.d.'s in cell parameters are only used when they are defined by crystal symmetry. An approximate (isotropic) treatment of cell e.s.d.'s is used for estimating e.s.d.'s involving l.s. planes.
Refinement. Refinement of *F*^2^ against ALL reflections. The weighted *R*-factor *wR* and goodness of fit *S* are based on *F*^2^, conventional *R*-factors *R* are based on *F*, with *F* set to zero for negative *F*^2^. The threshold expression of *F*^2^ > σ(*F*^2^) is used only for calculating *R*-factors(gt) *etc*. and is not relevant to the choice of reflections for refinement. *R*-factors based on *F*^2^ are statistically about twice as large as those based on *F*, and *R*- factors based on ALL data will be even larger.

### Fractional atomic coordinates and isotropic or equivalent isotropic displacement parameters (Å^2^)

**Table:**

	*x*	*y*	*z*	*U*_iso_*/*U*_eq_	
S1	1.24481 (17)	0.73593 (5)	0.74840 (3)	0.01960 (16)	
F1	−0.1405 (4)	0.22456 (12)	0.90756 (7)	0.0262 (4)	
O1	0.7062 (4)	0.74520 (13)	0.92210 (7)	0.0173 (4)	
N1	0.3500 (6)	0.63660 (18)	0.97059 (10)	0.0169 (5)	
N2	0.6774 (5)	0.54684 (16)	0.83437 (9)	0.0146 (5)	
N3	0.8668 (5)	0.63110 (18)	0.81930 (9)	0.0143 (5)	
N4	1.0264 (6)	0.5213 (2)	0.74068 (10)	0.0181 (5)	
C1	0.2029 (7)	0.5313 (2)	0.96080 (11)	0.0149 (6)	
C2	−0.0082 (7)	0.4801 (2)	0.99618 (11)	0.0173 (6)	
H2A	−0.0732	0.5149	1.0324	0.021*	
C3	−0.1231 (7)	0.3755 (2)	0.97690 (12)	0.0180 (6)	
H3A	−0.2699	0.3370	0.9999	0.022*	
C4	−0.0221 (7)	0.3275 (2)	0.92389 (12)	0.0188 (7)	
C5	0.1898 (7)	0.3770 (2)	0.88805 (11)	0.0171 (6)	
H5A	0.2550	0.3412	0.8521	0.021*	
C6	0.3043 (6)	0.48216 (19)	0.90700 (11)	0.0131 (6)	
C7	0.5237 (6)	0.5616 (2)	0.88265 (11)	0.0124 (6)	
C8	0.5425 (7)	0.6604 (2)	0.92589 (11)	0.0138 (6)	
C9	1.0386 (6)	0.6223 (2)	0.76802 (11)	0.0153 (6)	
C10	1.1774 (7)	0.4928 (2)	0.68493 (11)	0.0205 (7)	
H10A	1.2888	0.4200	0.6901	0.025*	
H10B	1.3211	0.5544	0.6751	0.025*	
C11	0.9584 (7)	0.4796 (2)	0.63365 (12)	0.0211 (7)	
H11A	1.0663	0.4636	0.5965	0.032*	
H11B	0.8445	0.5509	0.6291	0.032*	
H11C	0.8232	0.4157	0.6423	0.032*	
H1N1	0.326 (7)	0.679 (2)	1.0006 (11)	0.026 (9)*	
H1N3	0.866 (6)	0.696 (2)	0.8394 (10)	0.009 (7)*	
H1N4	0.928 (7)	0.473 (2)	0.7566 (12)	0.018 (8)*	

### Atomic displacement parameters (Å^2^)

**Table:**

	*U*^11^	*U*^22^	*U*^33^	*U*^12^	*U*^13^	*U*^23^
S1	0.0212 (4)	0.0175 (3)	0.0201 (3)	−0.0010 (3)	0.0025 (3)	0.0015 (3)
F1	0.0337 (11)	0.0178 (7)	0.0269 (9)	−0.0080 (8)	0.0005 (8)	0.0005 (7)
O1	0.0213 (12)	0.0156 (8)	0.0150 (9)	−0.0033 (9)	0.0001 (8)	−0.0028 (7)
N1	0.0218 (14)	0.0167 (11)	0.0121 (12)	0.0002 (11)	0.0024 (11)	−0.0060 (10)
N2	0.0146 (14)	0.0166 (10)	0.0124 (11)	0.0004 (10)	−0.0012 (10)	0.0009 (9)
N3	0.0157 (13)	0.0150 (10)	0.0121 (12)	0.0007 (10)	0.0019 (10)	−0.0023 (10)
N4	0.0214 (15)	0.0194 (11)	0.0136 (13)	−0.0022 (11)	0.0043 (11)	−0.0024 (10)
C1	0.0178 (17)	0.0140 (11)	0.0130 (13)	0.0044 (12)	−0.0019 (12)	0.0020 (10)
C2	0.0191 (17)	0.0203 (13)	0.0125 (14)	0.0041 (13)	0.0013 (13)	0.0018 (11)
C3	0.0164 (16)	0.0199 (13)	0.0176 (15)	0.0002 (12)	−0.0001 (12)	0.0089 (12)
C4	0.0226 (18)	0.0130 (12)	0.0209 (15)	0.0001 (13)	−0.0061 (13)	0.0021 (12)
C5	0.0198 (17)	0.0161 (12)	0.0153 (14)	0.0006 (12)	−0.0028 (12)	−0.0008 (11)
C6	0.0149 (16)	0.0140 (11)	0.0105 (13)	0.0030 (11)	0.0004 (11)	0.0020 (10)
C7	0.0148 (16)	0.0138 (12)	0.0085 (13)	0.0030 (11)	−0.0020 (11)	−0.0001 (10)
C8	0.0147 (16)	0.0165 (12)	0.0103 (13)	0.0041 (12)	−0.0033 (11)	−0.0010 (11)
C9	0.0137 (15)	0.0203 (13)	0.0118 (13)	0.0038 (12)	−0.0015 (11)	0.0024 (11)
C10	0.0234 (18)	0.0204 (13)	0.0179 (15)	0.0000 (13)	0.0063 (13)	−0.0042 (11)
C11	0.0266 (19)	0.0200 (13)	0.0166 (15)	−0.0022 (14)	0.0043 (13)	−0.0021 (12)

### Geometric parameters (Å, º)

**Table:**

S1—C9	1.673 (3)	C2—C3	1.390 (4)
F1—C4	1.359 (3)	C2—H2A	0.9500
O1—C8	1.234 (3)	C3—C4	1.385 (4)
N1—C8	1.352 (3)	C3—H3A	0.9500
N1—C1	1.409 (3)	C4—C5	1.373 (4)
N1—H1N1	0.84 (2)	C5—C6	1.392 (3)
N2—C7	1.293 (3)	C5—H5A	0.9500
N2—N3	1.342 (3)	C6—C7	1.459 (4)
N3—C9	1.387 (3)	C7—C8	1.501 (3)
N3—H1N3	0.88 (2)	C10—C11	1.521 (4)
N4—C9	1.323 (3)	C10—H10A	0.9900
N4—C10	1.457 (3)	C10—H10B	0.9900
N4—H1N4	0.80 (3)	C11—H11A	0.9800
C1—C2	1.373 (4)	C11—H11B	0.9800
C1—C6	1.406 (3)	C11—H11C	0.9800
			
C8—N1—C1	111.5 (2)	C5—C6—C1	119.6 (2)
C8—N1—H1N1	123 (2)	C5—C6—C7	133.9 (2)
C1—N1—H1N1	125 (2)	C1—C6—C7	106.4 (2)
C7—N2—N3	117.0 (2)	N2—C7—C6	126.2 (2)
N2—N3—C9	120.7 (2)	N2—C7—C8	127.4 (2)
N2—N3—H1N3	119.8 (17)	C6—C7—C8	106.4 (2)
C9—N3—H1N3	119.0 (17)	O1—C8—N1	126.8 (2)
C9—N4—C10	125.2 (2)	O1—C8—C7	126.8 (2)
C9—N4—H1N4	116.1 (19)	N1—C8—C7	106.4 (2)
C10—N4—H1N4	118.7 (19)	N4—C9—N3	115.0 (2)
C2—C1—C6	122.8 (2)	N4—C9—S1	126.9 (2)
C2—C1—N1	127.8 (2)	N3—C9—S1	118.02 (19)
C6—C1—N1	109.3 (2)	N4—C10—C11	111.2 (2)
C1—C2—C3	117.3 (2)	N4—C10—H10A	109.4
C1—C2—H2A	121.3	C11—C10—H10A	109.4
C3—C2—H2A	121.3	N4—C10—H10B	109.4
C4—C3—C2	119.6 (3)	C11—C10—H10B	109.4
C4—C3—H3A	120.2	H10A—C10—H10B	108.0
C2—C3—H3A	120.2	C10—C11—H11A	109.5
F1—C4—C5	119.1 (2)	C10—C11—H11B	109.5
F1—C4—C3	116.9 (3)	H11A—C11—H11B	109.5
C5—C4—C3	124.0 (2)	C10—C11—H11C	109.5
C4—C5—C6	116.7 (2)	H11A—C11—H11C	109.5
C4—C5—H5A	121.7	H11B—C11—H11C	109.5
C6—C5—H5A	121.7		
			
C7—N2—N3—C9	−179.6 (2)	N3—N2—C7—C8	−3.2 (4)
C8—N1—C1—C2	−179.4 (3)	C5—C6—C7—N2	−2.3 (5)
C8—N1—C1—C6	0.4 (3)	C1—C6—C7—N2	177.7 (3)
C6—C1—C2—C3	−0.1 (4)	C5—C6—C7—C8	179.8 (3)
N1—C1—C2—C3	179.7 (2)	C1—C6—C7—C8	−0.1 (3)
C1—C2—C3—C4	0.1 (4)	C1—N1—C8—O1	−178.9 (2)
C2—C3—C4—F1	179.4 (2)	C1—N1—C8—C7	−0.4 (3)
C2—C3—C4—C5	0.2 (4)	N2—C7—C8—O1	1.0 (5)
F1—C4—C5—C6	−179.8 (2)	C6—C7—C8—O1	178.8 (2)
C3—C4—C5—C6	−0.6 (4)	N2—C7—C8—N1	−177.5 (3)
C4—C5—C6—C1	0.6 (4)	C6—C7—C8—N1	0.3 (3)
C4—C5—C6—C7	−179.4 (3)	C10—N4—C9—N3	177.4 (2)
C2—C1—C6—C5	−0.3 (4)	C10—N4—C9—S1	−4.7 (4)
N1—C1—C6—C5	179.9 (2)	N2—N3—C9—N4	−7.8 (4)
C2—C1—C6—C7	179.7 (3)	N2—N3—C9—S1	174.10 (18)
N1—C1—C6—C7	−0.2 (3)	C9—N4—C10—C11	−109.6 (3)
N3—N2—C7—C6	179.4 (2)		

### Hydrogen-bond geometry (Å, º)

**Table:**

*D*—H···*A*	*D*—H	H···*A*	*D*···*A*	*D*—H···*A*
N1—H1*N*1···O1^i^	0.84 (2)	2.01 (2)	2.836 (3)	168 (3)
N3—H1*N*3···O1	0.88 (2)	2.06 (2)	2.747 (3)	134 (2)
N4—H1*N*4···S1^ii^	0.80 (3)	2.86 (2)	3.541 (2)	144 (2)

Symmetry codes: (i) *x*−1/2, −*y*+3/2, −*z*+2; (ii) −*x*+2, *y*−1/2, −*z*+3/2.
